# Medial Open‐wedge Osteotomy with Double‐plate Fixation for Varus Malunion of the Distal Femur

**DOI:** 10.1111/os.12421

**Published:** 2019-02-05

**Authors:** Qi‐fang He, Han‐xu Wang, Hui Sun, Yu Zhan, Bin‐bin Zhang, Xue‐tao Xie, Cong‐feng Luo

**Affiliations:** ^1^ Department of Orthopaedic Surgery Shanghai Jiao Tong University Affiliated Sixth People's Hospital Shanghai China

**Keywords:** Distal femur fractures, Double plate, Medial open‐wedge osteotomy, Varus malunion

## Abstract

**Objective:**

To present our clinical experience of treating varus malunion of the distal femur through a medial open‐wedge osteotomy with double‐plate fixation.

**Methods:**

A prospective cohort study was performed. From January 2005 to February 2015, 15 consecutive patients with varus malunion following distal femur fractures were surgically treated at a single level I trauma center. The coronal and sagittal deformity were corrected by a medial open‐wedge osteotomy of the distal femur. A medial buttress plate was used to maintain the realignment. A lateral locking plate was additionally used as a protection plate. The mean age of patients at the time of the surgery was 35.5 years (range, 22–58 years). The radiographical evaluation included the mechanical femorotibial angle, the mechanical lateral distal femoral angle, the anatomic posterior distal femoral angle, and the leg length discrepancy. Clinical outcome evaluation consisted of the range of motion (ROM) and Hospital for Special Surgery (HSS) score.

**Results:**

Mean follow‐up was 7.4 years (range, 4–11.5 years). Varus and flexion malalignment and limb discrepancy were adequately corrected in all patients. The mechanical femorotibial angle, the mechanical lateral distal femoral angle, and the anatomic posterior distal femoral angle were restored from 17.5° (range, 13°–25°) to 2.3° (range, − 2°–7°), 102.3° (range, 95°–112°) to 85.2° (range, 81°–92°), and 77.1° (range, 65°–87°) to 82.7° (range, 76°–88°), respectively. The leg length discrepancy was diminished from 3.4 cm (range, 2.4–4.5 cm) to 0.8 cm (range, 0–1.7 cm). The average bone healing time was 4.1 months (range, 2.5–6 months). The average ROM of the affected knees at 24‐month follow‐up was 3.4°–112.55°. The score of HSS at 4‐years follow‐up was 76.1 (range, 64–88). No internal fixation failure or secondary operation was noted until the last follow‐up.

**Conclusion:**

Medial open‐wedge osteotomy can adequately correct the posttraumatic varus malunion of the distal femur. With fixation of the double plate, non‐displaced bone healing and good functional outcome are expected.

## Introduction

Distal femur fractures (DFF) account for 3%–6% of femoral fractures^1^. Patients with these injuries generally present with a bimodal distribution: young patients after high‐energy trauma and elderly patients after low‐energy falls from standing height^2^. Treating comminuted or geriatric DFF is challenging for traumatologists. Complications are seen throughout the published literature, including implant failure, malunion, and nonunion^1^, ^3^, ^4^, ^5^. Unreliable fixator and mal‐reduction are associated with intraoperative or postoperative varus collapse of the distal fragment^6^. The resulting varus malunion, often accompanied by flexion deformity of the distal femur and limb shortening, may be increased by the quadriceps, hamstring, and adductor muscle groups^1^. Davison reports more than 5° of varus collapse to occur in 42% of the comminuted distal femur with the fixation of lateral condylar buttress plate^7^. Despite the popularity of the locking compression plate (LCP) and the Less Invasive Stabilization System (LISS) in the past decade, postoperative malunion is still reported^3^, ^8^.

Conservative therapy is not effective in managing this post‐traumatic deformity. To restore the function of joint maximally, surgical correction of the displaced mechanical axis is needed^9^, ^10^. Different distal femoral osteotomies have been proposed. However, the shortcomings of different osteotomies have remained obvious and there is no consensus about the most appropriate strategy^10^, ^11^, ^12^, ^13^. Medial open‐wedge distal femoral osteotomy (OW‐DFO) is an anatomically corrective procedure for varus deformity. However, the rigidity provided by fixation of the single medial plate is likely insufficient in the absence of bone contact in the osteotomy gap, which may lead to delayed union or secondary failure of fixation^10^. Recently, the literature on the double‐plate technique has been growing, especially on treating comminuted and osteoporotic DFF^6^, ^14^, ^15^, ^16^. This fixation strategy provides a more rigid construct for the distal femur and may be utilized to stabilize an open‐wedge osteotomy.

We hypothesize that an OW‐DFO with the fixation of bilateral plates will improve the outcome of varus malunion of the distal femur. The aims of the present study are: (i) to determine the efficiency of this strategy on the deformity correction; (ii) to investigate the duration of bone healing; and (iii) to evaluate the functional outcome of patients.

## Materials and Methods

### *Inclusion and Exclusion Criteria*

Patients who met the following criteria were included: (i) age >18 years; (ii) with a history of DFF and previous surgical treatment; (iii) varus malunion after previous surgery; and (iv) had provided consent to accept OW‐DFO and varus malunion after a DFF. Patients were excluded based on following criteria: (i) malunited pathological fractures; (ii) prior open fractures with neurovascular injury; and (iii) concomitant intra‐articular deficit, rotational deformity of the distal femur or deformity of the proximal tibia.

### *Patients*

From January 2005 to February 2015, 15 consecutive patients were surgically treated at a single level I trauma center. The malunion was corrected through the OW‐DFO, and fixed with bilateral plates of the distal femur. All included patients were operated on by the corresponding author. The postoperative radiological and clinical outcome were assessed. We performed a prospective cohort study. Approval from the institution's ethical review board was obtained prior to the initiation of the study.

### *Preoperative Planning*

Patients underwent a standard radiologic protocol of standard and full‐length standing anteroposterior radiographs, CT scans, and image reconstructions after admission. The radiological data were evaluated through Picture Archiving and Communication Systems (PACS), including the mechanical femorotibial angle (mFTA), the mechanical lateral distal femoral angle (mLDFA), the anatomic posterior distal femoral angle (aPDFA), and leg length discrepancy (LLD). CT scans and image reconstructions were used to confirm that no intra‐articular deficit and significant rotational deformity existed.

#### *mFTA*

**Figure 1:**
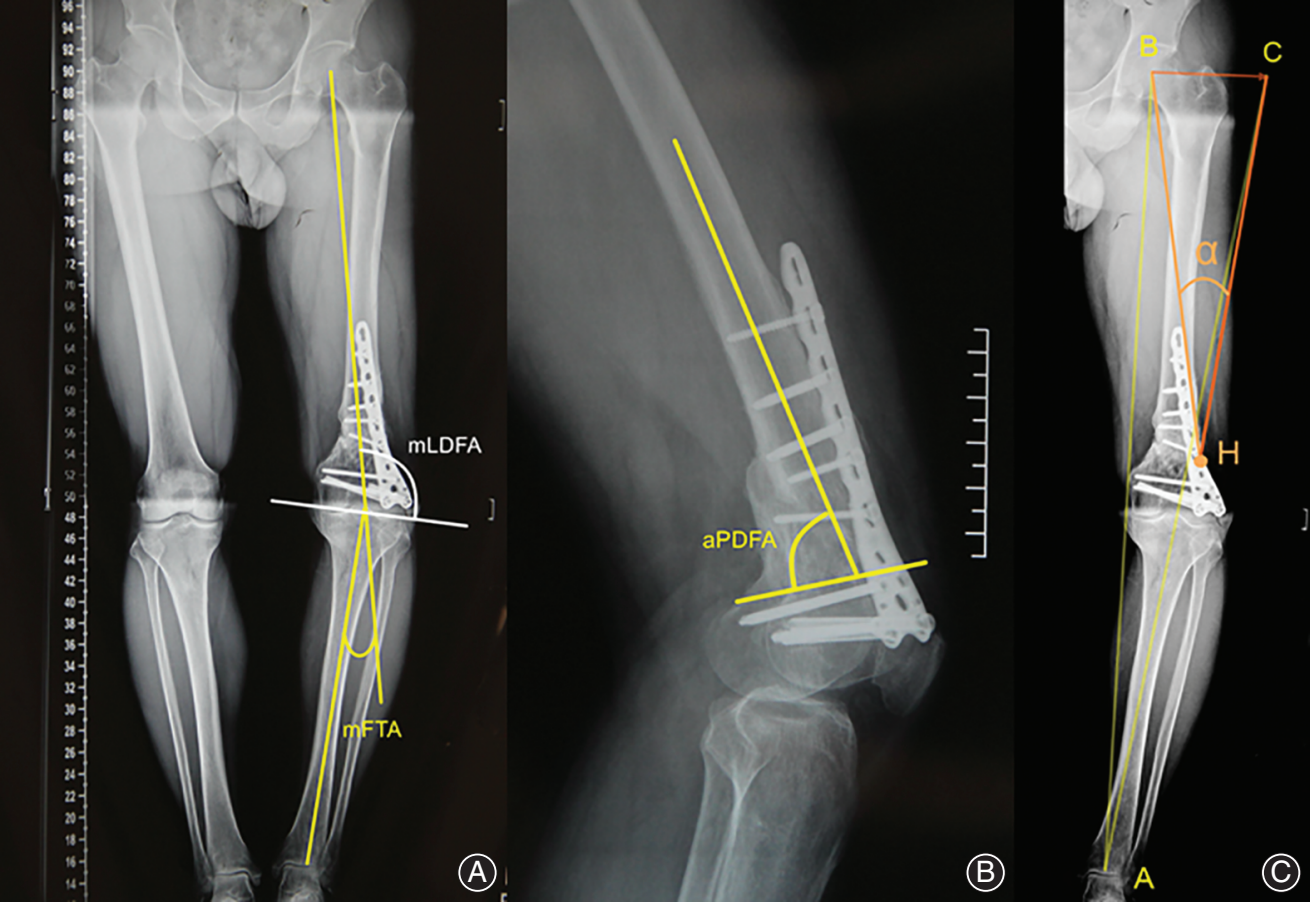
The preoperative evaluation and planning. (A) The mechanical femorotibial angle (mFTA) is measured from long‐limb X‐rays. (B) The anatomic posterior distal femoral angle (aPDFA) is measured from the lateral view of femur. (C) The expected correction angle (α) is evaluated using the Miniaci method. The current mechanical axis is a line connecting the center of the femoral head (B) with the center of the upper ankle joint (A). Position C is at the level of point B on a line starting from point A through the center of knee. The expected mechanical axis is the line AC. Position H is the hinge point of the open‐wedge osteotomy. The angle between the lines HB and HC corresponds to the correction angle at the distal femur.

mFTA is defined as the angle formed by the mechanical axis of the femur and tibia (Fig. 1a). An mFTA of 0° indicate a neutral mechanical axis of the limb without valgus or varus deformity. An increased mFTA suggests a varus deformity of the limb, and decreased mFTA suggests a valgus deformity.

#### *mLDFA*

The lateral angle formed by the mechanical axis of the femur and knee joint line was defined as the mLDFA, which is an indicator of coronal alignment of the distal femur (Fig. 1a). The standard value of mLDFA is 87° ± 3°. An increased mLDFA value indicates varus deformity of the distal femur. Conversely, a decreased mLDFA indicates a valgus malalignment.

#### *aPDFA*

aPDFA is the angle between the anatomic axis and the sagittal distal femoral joint orientation line, which is used to describe the sagittal alignment of the femur (Fig. 1b). The aPDFA is measured from the lateral view of the femur, with a normal mean value of 83° (range: 79°–87°)^17^.

#### *LLD*

According to Sanjeev's research, limb length discrepancy is evaluated from the full‐length standing anteroposterior radiograph^18^. The length of the lower limb was measured from the proximal end of the femoral head to the center of the tibial plafond on each side and the difference (LLD) was calculated in millimeters.

#### *Correction Angle (α)*

The expected correction angle (α) is evaluated using the Miniaci method (Fig. 1c). This angle α is projected at the medial side of the distal femur. The height of the osteotomy gap can then be measured taking into account the magnification factor.

### *Surgical Technique*

#### *Patient Position and Approach*

General anesthesia is used with the patient supine on a radiolucent table and a bump placed under the buttock to maintain the leg in a neutral rotational position. The entire limb, including the iliac crest, is prepared and draped free before a sterile tourniquet is applied. Besides the original lateral incision, a medial approach is used.

#### *Osteotomy*

**Figure 2:**
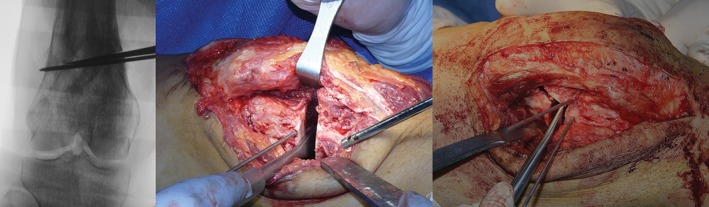
The single plane and biplanar osteotomy. (A) The osteotome is inserted into the gap slowly to preserve approximately 1 cm of a lateral bone bridge as a hinge. (B) A single plane osteotomy is performed. (C) Biplanar osteotomy creates more potential bony contact.

If the failed lateral implants remain, the surgery starts with the removal of the implants from the original lateral approach. The incision is closed temporarily afterward. A 12‐cm straight‐line incision is made on the medial side, starting from the medial joint line and ascending along the posterior border of the adductor magnus tendon. After the division of the subcutaneous tissue and the fasciotomy, the tendon of the adductor magnus and the vastus medialis are retracted to the dorsal and ventral side, respectively. The distal femur is exposed sufficiently for the osteotomy and plate fixation. Two blunt Hohmann retractors are positioned anteriorly and posteriorly, exposing the anteromedial aspect of the supracondylar area and protecting the neurovascular structures of the posterior side of the femur. The hinge point is located proximal to the upper margin of the lateral femur condyle 5–10 mm from the lateral cortex. Two 2.3‐mm Kirschner wires are drilled into the medial supracondylar area toward the hinge point. The osteotomy is performed with an oscillating saw and osteotomes following the guide of the Kirschner wires. Approximately 1 cm of a lateral bone bridge is preserved as a hinge when either one plane or biplanar osteotomy is performed (Fig. 2). The osteotomy gap is then opened with an osteotomy spreader. The medial open‐wedge tibial osteotomy allows for realignment of the sagittal deformity of the distal femur with the eccentric distraction of the osteotomy gap. In correcting the flexion deformity, the osteotomy gap tends to open more posteriorly during spreading, decreasing the forward angle of the distal femur.

#### *Fixation of double plates*

**Figure 3:**
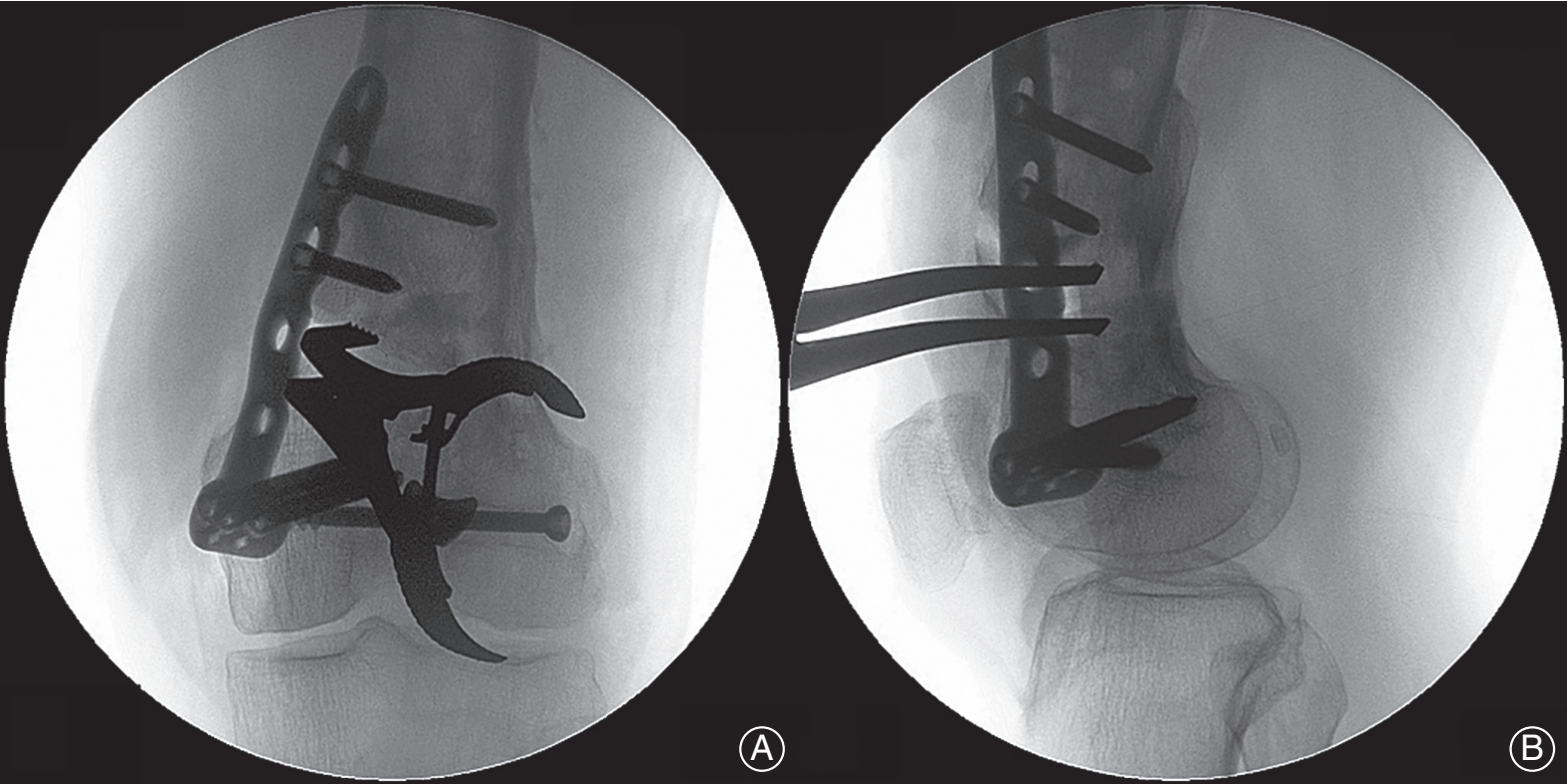
The intraoperative confirmation of correction. (A) The expected correction angle is examined through anteroposterior view. (B) To modulate the sagittal alignment, the spreader is adjusted to open the gap more posteriorly.

**Figure 4:**
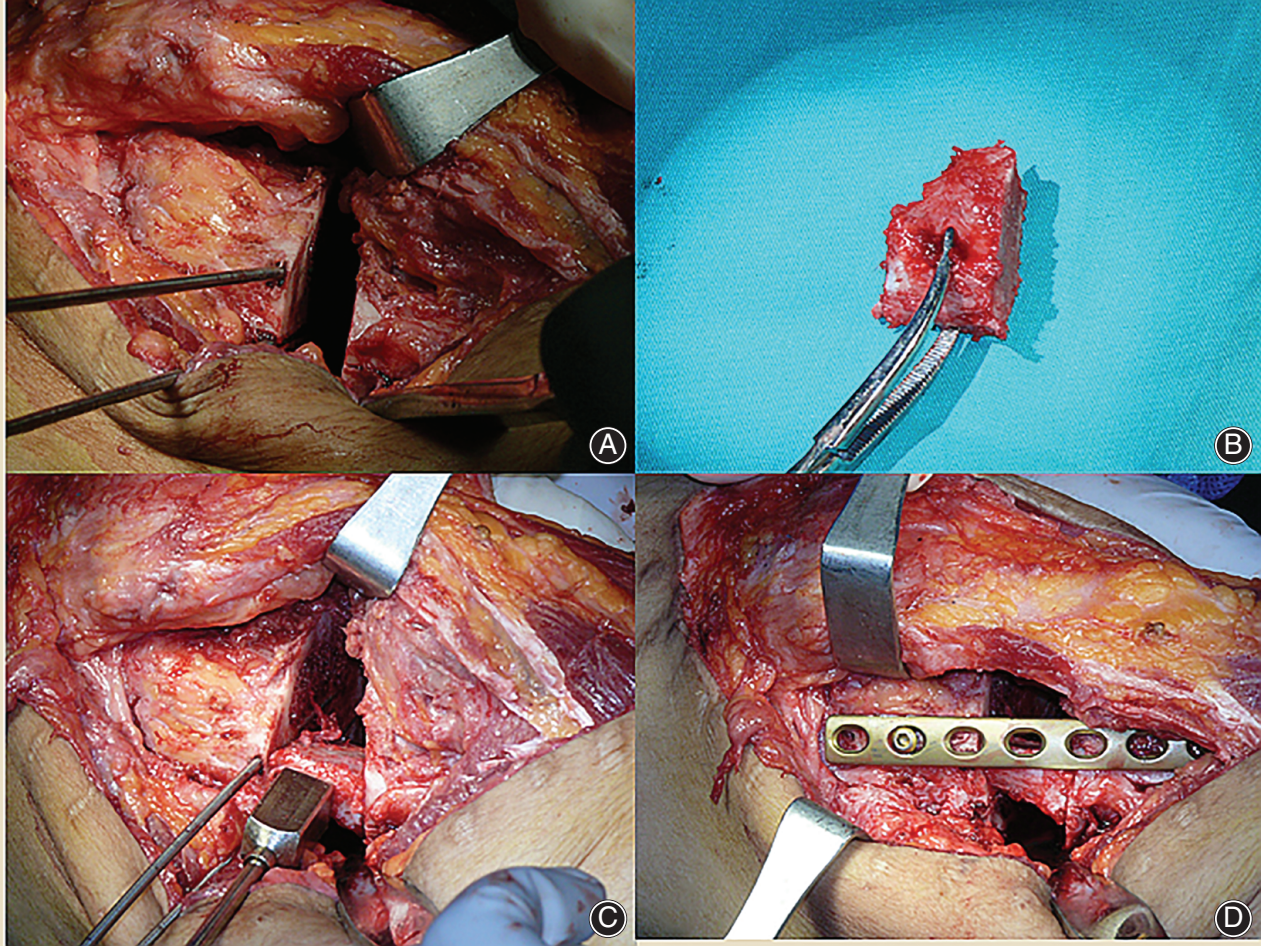
Iliac crest autograft is transplanted into the gap. Bicortical iliac crest autograft is harvested and cut to shape fitting the gap (A, B). The medial plate is fixed after insertion of the autograft (C, D).

Once the expected length of the open wedge is obtained, a 3.5‐mm 6‐hole or 8‐hole locking compression plate (LCP) is then placed medially to buttress the opened gap, with at least two screws on each side of the osteotomy. The alignment is confirmed fluoroscopically after planned correction is completed (Fig. 3). Iliac crest autograft or bicortical allograft is transplanted before the medial incision is temporarily closed (Fig. 4).

**Figure 5:**
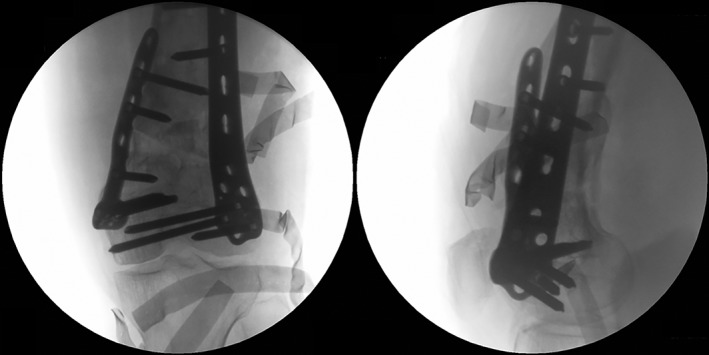
The reconfirmation after bilateral plates fixation. The osteotomy gap, aPDFA, plates position and condition of screws are checked.

Reopening the lateral approach, a 10‐hole or longer LCP or LISS is fixed over the periosteum. At least six cortices on each side of the osteotomy are fixed. The alignments in both planes are reconfirmed and recorded through image intensifier (Fig. 5).

Overflow drainage is recommended before the closure of the bilateral subcutaneous tissue and incisions. The X‐ray performance is assessed on the day following the osteotomy.

### *Postoperative Management*

Passive range‐of‐motion (ROM) exercises were started at the first postoperative day if pain was tolerated. With the help of a therapist or orthopedic doctor, toe‐touch weight‐bearing was allowed after the drainage was removed (usually 2 days after operation), and the weight was limited within 15–20 kilograms (Kg). Weight‐bearing was permitted according to the findings of radiographic and clinical examinations at the 8‐week follow‐up. Any exercise resulting in excessive load to the knee joint (i.e. walking up the stairs or full squatting) was not permitted until bone healing.

### *Radiological Assessment*

Mal‐reduction was defined as mLDFA >90° or <84°, or aPDFA >87° or <79°. Secondary loss of reduction was defined as an increase of more than 5° of the mLDFA and aPDFA compared with the first preoperative X‐ray observation. Long‐limb films were obtained at 6 months after the operation, from which the mFTA was measured. The osteotomy was considered as radiographically united when three cortices of the bone were united on the anteroposterior (AP) and lateral views of the bone. Nonunion was defined if 3 consecutive months’ X‐rays did not show progressive healing.

### *Clinical Assessment*

The pain of patients was evaluated using the visual analogue scale (VAS). Functional outcome evaluation included ROM and Hospital for Special Surgery (HSS) score. Patients who accepted revision or secondary total knee arthroplasty (TKA) were recorded.

### *Statistical Methods*

SPSS Statistics 19.0 software (IBM SPSS, Chicago, IL, US) was used for the statistical analyses. The descriptive statistics were used to determine ranges, means, and standard deviations. Student paired *t*‐tests were used to compare radiographic measurements. A *P*‐value <0.01 was considered significant.

## Results

The 15 consecutive patients consisted of 5 women and 10 men. The mean age of patients was 35.5 years (range, 22–58 years). Mean follow‐up was 7.4 years (range, 4–11.5 years) (Table 1).

**Table 1:** Information about patients

Patients (number)	Gender/Age (years)	Body weight	Fracture type	Previous implant	Follow‐up (years)
1	M/41	Normal	A3	IM (removed)	5.5
2	F/26	Normal	B2	LISS (removed)	4.5
3	F/58	Obese	B2	CBP (removed)	9.5
4	M/22	Normal	A3	LC‐DCP (removed)	9.5
5	M/26	Normal	A2	LCP (remained)	10
6	M/20	Obese	A3	IM (removed)	10
7	F/32	Over	C3	LCP (removed)	11
8	M/20	Normal	A3	DCS (removed)	11.5
9	M/47	Over	A3	CBP (removed)	10
10	M/36	Normal	A3	LCP (remained)	4
11	F/43	Over	A3	IM (removed)	9
12	M/46	Normal	C3	LCP (remained)	6
13	M/27	Normal	B2	LCP (removed)	5
14	F/46	Normal	A3	LC‐DCP (removed)	5
15	M/23	Normal	A3	LCP (remained)	5

CBP, condylar buttress plate; DCS, dynamic condylar screw; F, female; IM, intramedullary nail; LC‐DCP, limited contact dynamic compression plate; M, male.

### *Operation and Complications*

Mean operation time was 110.5 min (range, 95–144 min). Blood loss was 307.2 mL (range, 209–412 mL). No iatrogenic nerve and vessels damage were noted. Two cases of superficial infections healed after closed irrigation. In 2 cases in this study, hinges broke during the operation. An autologous bicortical iliac graft was used in 11 cases, and allograft was used in 4 cases. No mal‐reduction was observed.

### *Radiological Results*

The outcomes of corrections are shown in Table 2.

**Table 2:** The outcome of correction

Patients (number)	mLDFA (pre‐/post‐)*	aPDFA (pre‐/post‐)*	LLD (cm)	mFTA (pre‐/6 months)†
1	104°/88°	65°/76°	3.5/0.7	18°/3°
2	103°/91°	86°/85°	4.1/1.3	18°/4°
3	96°/84°	76°/84°	3.2/0.6	15°/−2°
4	106°/82°	71°/85°	3.1/0.4	17°/2°
5	105°/91°	75°/76°	4.5/1.0	25°/6°
6	98°/83°	80°/81°	3.3/0.8	24°/5°
7	99°/81°	81°/81°	2.8/1.3	17°/−1°
8	95°/85°	81°/88°	2.4/1.2	14°/2°
9	100°/87°	80°/79°	3.1/0.4	17°/4°
10	97°/80°	75°/84°	3.2/0	13°/−3°
11	109°/87°	79°/83°	2.9/1.0	14°/−1°
12	107°/81°	87°/86°	3.7/0.5	13.4°/0°
13	110°/84°	74°/80°	3.1/1.1	17°/5°
14	100°/92°	77°/84°	3.3/0.6	15°/3°
15	103°/82°	70°/89°	4.5/1.7	25°/7°

* post‐, the postoperative evaluation from the X‐ray of the next day of the operation; pre‐, preoperative evaluation of X‐ray

† The postoperative evaluation of mFTA was performed from the long limb X‐ray at 6 months after the operation.

#### *mFTA*

The average preoperative mFTA was significantly corrected from 17.5° (range, 13°–25°) preoperatively to 2.3° (range, − 2°–7°) postoperatively (*P* = 0.003).

#### *mLDFA*

The average mLDFA was improved from 102.3° (range, 95°–112°) preoperatively to 85.2° (range, 81°–92°) postoperatively (*P*= 0.002).

#### *aPDFA*

The average aPDFA was corrected from 77.1° (range, 65°–87°) preoperatively to 82.7° (range, 76°–88°) postoperatively (*P* = 0.002).

#### *LLD*

The average preoperative LLD was 3.38 cm (range, 2.4–4.1 cm). The LLD was significantly diminished to 0.8 cm (range, 0–1.7 cm) postoperatively (*P* = 0.001).

#### *Bone healing*

The average bone healing time was 4.1 months (range, 2.5–6 months); 61.9% (13/21) of patients achieved radiographic bony union in 3 months, and 38.1% (8/21) achieved bony union within 3 and 6 months. No nonunion or fixation failure was noted.

#### *Clinical Results*

All patients initiated full weight‐bearing within 3 months. Preoperative mean VAS score and HSS score were 37.1 (range, 18–74) and 42.5 (range, 28–61).

**Figure 6:**
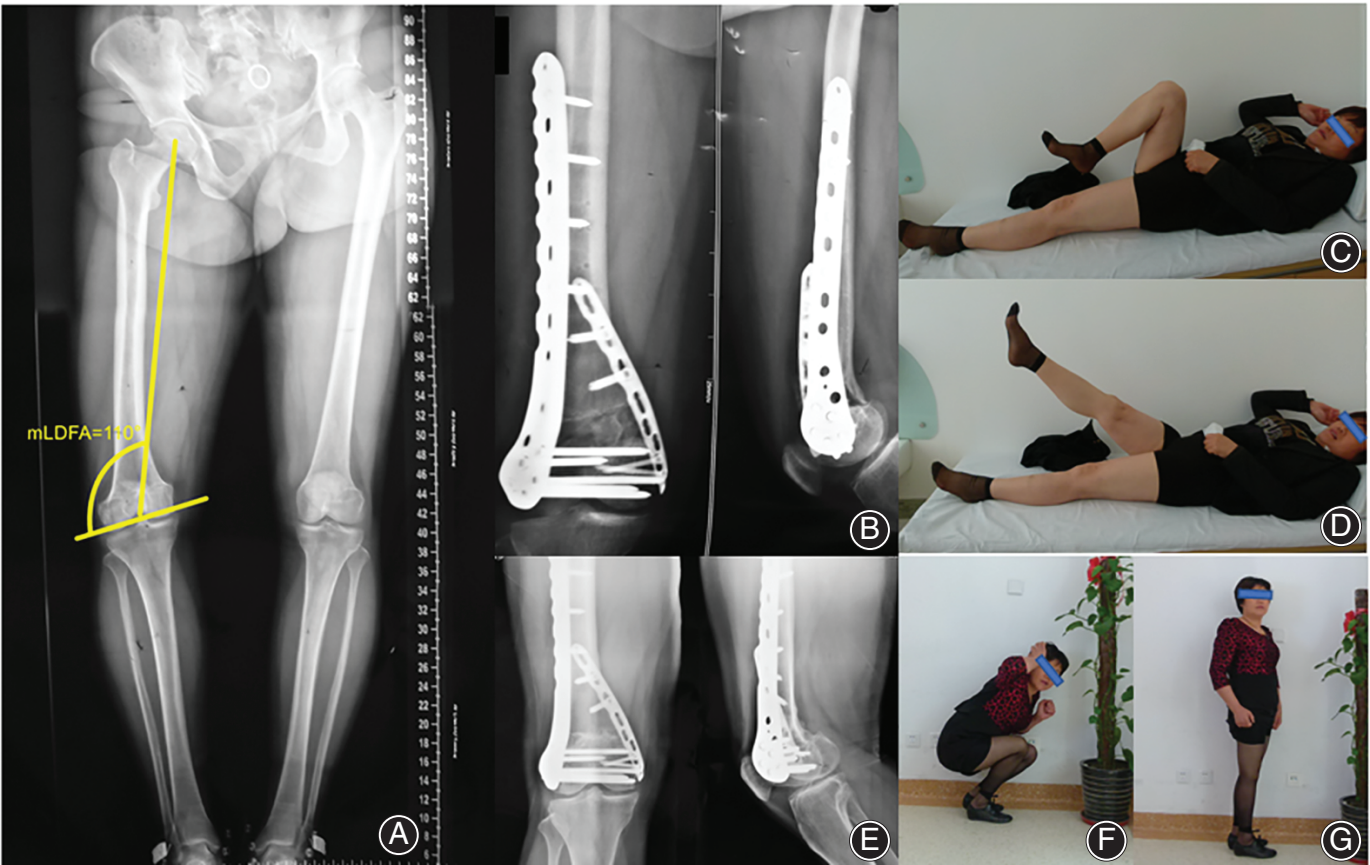
A female patient treated with medial open‐wedge distal femoral osteotomy (OW‐DFO) and double‐plate fixation. The preoperative X‐ray demonstrated a varus deformity after conservative treatment of previous distal femoral fracture (A). At 3‐month follow‐up, the osteotomy gap is filled with callus, and a satisfying range of motion was obtained (B, C, D). The patient presented with a further improved functional outcome at 2‐year follow‐up (E, F, G).

**Figure 7:**
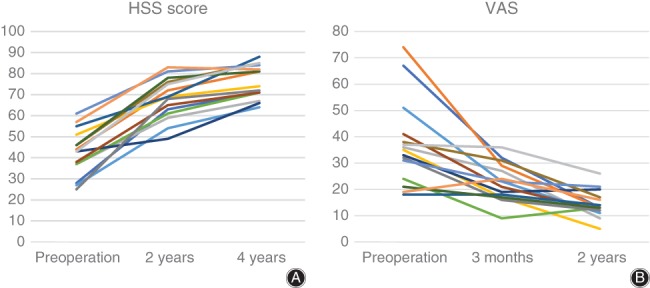
(A) The visual analogue scale (VAS) score and (B) Hospital for Special Surgery (HSS) score. The VAS dropped from 37.3 (preoperation) to 14.3 (24 months after operation). The average HSS increased from 42.5 (preoperation) to 76.1 (4 years after operation).

The average ROM of the affected knees at 24‐month follow up was 3.4°–112.55° (Fig. 6). The VAS score and HSS were improved after surgery (Fig. 7). No patient accepted secondary revision or TKA.

## Discussion

During the weight‐bearing status, a varus medial femoral condyle exerts excessive compressive stress on the medial tibiofemoral joint, which will accelerate degeneration of the joint and lead to post‐traumatic osteoarthritis (PTOA) eventually^19^, ^20^, ^21^. TKA is indicated for posttraumatic deformity with symptomatic PTOA, which can simultaneously manage the deformity as well as cartilage deficit^22^. Especially for elderly patients, an immediate improvement of symptoms and functional outcomes is expected. However, implementation of a TKA in patients with PTOA and limb deformity is technically challenging. Extended operation time and implant systems with higher constraint and modular options are often required^23^, ^24^, ^25^. Patients undergoing a TKA for PTOA often have increased rates of revision, postoperative infection, additional procedures, and complications compared with patients undergoing a TKA for primary osteoarthritis^26^, ^27^. Importantly, many patients with malunion of the distal femur are relatively younger, and the life expectancy of prostheses remains a major concern.

There is a renewed interest in joint preservation surgery. Closed‐wedge osteotomy has a high success rate and contributes to early weight‐bearing. Lobenhoffer reports that the medial closed wedge osteotomy of the distal femur healed in only 4–6 weeks, facilitating full weight‐bearing at 4 weeks after surgery^28^. In a report of lateral closed‐wedge osteotomy treating 16 cases of distal femoral varus deformity due to different causes, the mean bone healing time was 3 months^29^. One of the problems of close‐wedge osteotomy is the accuracy of correction. The surgeon is very reliant on the preoperative plan for the accuracy of bony resection; even so, precise resection of a wedge is technically difficult during surgery^30^. Besides, shortening of the limb will increase with the enlargement of the correction angle^13^. A closed wedge would enlarge the preexisting limb discrepancy, which is already a common source of dissatisfaction and litigation in patients with varus malunion^31^. A femoral supracondylar focal dome osteotomy reported recently could partly offset the limb shortening by rotating the distal femur frontally around an osteotomy dome^10^. Nonetheless, due to the absence of a hinge point, it is difficult to achieve a controlled correction on the sagittal deformity, which is frequently associated with varus malunion.

Medial open‐wedge distal femoral osteotomy is effective for medium or large corrections and is particularly easy to perform^32^, ^33^. Comparatively, the medial OW‐DFO could be a more anatomic restoration. The OW‐DFO technique allows fine‐tuning of deformity correction with the application of an opening device such as a laminar spreader until the desired angle is achieved. The sagittal correction can be achieved through rebalance of the anterior and posterior width of the gap during the correction of coronal deformity^34^. Simultaneously, the leg length discrepancy is minimized. Matsui reported a case of a medial opening wedge distal femoral osteotomy, through which multiplane deformity after DFF was corrected adequately and the leg length discrepancy was reduced from 32 mm to 5 mm^9^. OW‐DFO has no superiority over close‐wedge osteotomy. It is reported that open‐wedge osteotomy of proximal tibia is associated with a 4.3%–12% delayed union and 3%–5.4% non‐union^35^, ^36^, ^37^, ^38^, ^39^. One study of lateral OW‐DFO series reported 3/13 cases (13%) of delayed union^40^. Edgerton reports a relatively high incidence of nonunion (25%) and loss of correction (21%) after distal femoral varus osteotomy for painful genu valgum^12^. There is no related literature regarding the incidence of delayed union or nonunion after medial OW‐DFO, but in Matsui's case, it took 6 months to see the bridging callus fill the distal femoral osteotomy gap and 1 year to achieved an osseous union. Besides the risk of nonunion or fixator failure during postoperative exercise, longer healing time is adverse to the functional rehabilitation of patients.

This potential problem may be prevented by the double‐plate fixation, which has obtained encouraging outcomes in treating comminuted and geriatric fractures of the distal femur^6^, ^14^, ^15^, ^16^. Using a lateral locking plate with an additional medial buttress plate increased the fixation construct rigidity, facilitated graft impaction, and enabled early rehabilitation without loss of reduction^6^, ^41^. Compared with single plate fixation, double‐plate fixation provided better resistance to compression as well as bending and torsion in biomechanical tests^41^, ^42^. Steinberg (2017) treated 32 elderly patients for distal femoral fractures using the double‐plate technique. Full weight‐bearing was permitted as early as 7 weeks after the operation, and 93.7% (30/32) of fractures healed within 12 weeks with good axial alignment^16^. Following the same principle, in the present study, the medial buttress plate was used to maintain the width of the osteotomy gap, and the major instability created by osteotomy was counteracted by using the bulky lateral locking plate as a fixed‐angle device. Through the improvement of stability, scheduled weight‐bearing (8–12 weeks) was achieved and loss of correction was prevented. Fracture of the lateral cortical hinge has been reported to occur in 16% to 25% of patients after medial opening wedge high tibial osteotomy, which is recognized as a risk factor of prolonged bone healing time and nonunion^35^, ^37^, ^43^. Practically, it is unlikely for hinge fracture to be completely avoided, especially if there is an incidence of latent hinge disruption^39^. The 2 cases of hinge fracture in this study achieved bone union within 3 months, which may have been due to the reduced stability being compensated by the bilateral plates.

There are several concerns to note about this combined strategy. First, the position of the original incision should be considered when the medial approach is performed. Usually, a 5–7‐cm skin bridge should be preserved to avoid avascular necrosis of skin^44^. Second, a potential vascular injury to the medical aspects of the distal thigh may be expected during the procedure^45^. The medial superior genicular artery and the third perforating artery to the vastus medialis muscle supply the operative area. Meticulous dissection is needed and a smaller plate on the medial side is preferred. Third, the excessive stripping of the bilateral periosteum of the distal femur is detrimental to bone healing. The stripping of the medial periosteum should be restricted to the osteotomy area, and the lateral periosteum should be preserved.

There are several limitations to this study. First, despite its prospective nature, this study does not include a control group, which is practically difficult in a small sample study. Second, the preoperative soft tissue injuries were not listed. The reason is that, before the surgery, no sign of instability caused by dysfunction of ligaments was found in patients. Third, there is a lack of direct biomechanical evidence to support the importance of the double‐plate technique for medial OW‐DFO. Related research is under way.
